# Molecular Mechanisms Underlying the Link between Nuclear Receptor Function and Cholesterol Gallstone Formation

**DOI:** 10.1155/2012/547643

**Published:** 2011-11-01

**Authors:** Mary Carmen Vázquez, Attilio Rigotti, Silvana Zanlungo

**Affiliations:** ^1^Departamento de Gastroenterología, Escuela de Medicina, Pontificia Universidad Católica, Marcoleta No. 367, 8330024 Santiago, Chile; ^2^Departamento de Nutrición, Diabetes y Metabolismo, Escuela de Medicina, Pontificia Universidad Católica, Marcoleta No. 367, 8330024 Santiago, Chile

## Abstract

Cholesterol gallstone disease is highly prevalent in western countries, particularly in women and some specific ethnic groups. The formation of water-insoluble cholesterol crystals is due to a misbalance between the three major lipids present in the bile: cholesterol, bile salts, and phospholipids. Many proteins implicated in biliary lipid secretion in the liver are regulated by several transcription factors, including nuclear receptors LXR and FXR. Human and murine genetic, physiological, pathophysiological, and pharmacological evidence is consistent with the relevance of these nuclear receptors in gallstone formation. In addition, there is emerging data that also suggests a role for estrogen receptor ESR1 in abnormal cholesterol metabolism leading to gallstone disease. A better comprehension of the role of nuclear receptor function in gallstone formation may help to design new and more effective therapeutic strategies for this highly prevalent disease condition.

## 1. Introduction

Cholesterol gallstone disease (CGD) is one of the most common digestive disease conditions in both industrialized and developing western countries. Worldwide CGD prevalence ranges between 5% and 20% [1], being more common in women than men in every population that has been studied [2]. It is particularly prevalent in some specific ethnic groups including Mapuche and North American Indians as well as Chilean and Mexican Hispanics. Among these populations, CGD has an earlier onset and reaches prevalence rates over 50% and 70% in middle age male and women, respectively. CGD is also a key risk factor for gallbladder cancer. Therefore, CGD represents a serious burden for healthcare systems [3, 4].

Some of the pathogenic hallmarks of CGD are increased biliary cholesterol secretion, increased bile acid hydrophobicity, cholesterol microcrystal formation, growth, and aggregation with the formation of macroscopic stones in the gallbladder, and gallbladder inflammation [5–7]. The primary pathogenic mechanism associated with CGD is a disrupted balance between the three major lipids present in bile: cholesterol, bile salts, and phospholipids [8]. Under physiological conditions, bile cholesterol is kept in solution by its incorporation into mixed micelles together with phospholipids and bile salts. When either too much cholesterol or not enough solubilizing bile salt and phospholipid molecules are secreted, cholesterol comes out of solution and then crystallizes [9]. In addition, several biliary proteins have been described as nucleating factors that may promote cholesterol crystallization. Among them, there are immunoglobulins M and G, haptoglobin, *α*1-acid glycoprotein, aminopeptidase-N, *α*1-antichymotrypsin, and mucin. Despite correlative evidence between biliary levels and/or activity of these proteins and cholesterol precipitation in *in vitro* and animal models, only mucin seems to have a potential pathogenic role in human CGD [9]. Finally, impaired gallbladder motility is another important factor that contributes to further growth and aggregation of cholesterol microcrystals into macroscopic gallstones [7, 10].

In the hepatocyte, several types of proteins mediate the trafficking of lipids towards the canalicular pole for biliary secretion. These include multiple lipid transport-related gene products, lipoprotein receptors, basolateral lipid transporters, and intracellular lipid binding proteins as well as canalicular lipid transporters. Especially relevant for biliary lipid secretion and composition is the activity of ATP-binding-cassette- (ABC-) transport proteins expressed at the canalicular membrane. Among them, we can highlight the following ones: ABCB4, the transporter for phosphatidylcholine [11]; ABCB11, the bile salt export pump [12]; ABCG5/ABCG8, the obligate heterodimer that induces biliary cholesterol secretion [13].

Thus, biliary lipid secretion is controlled by a variety of proteins that mediate lipid uptake, transport, and metabolism in the liver. Furthermore, the expression of the genes encoding these proteins is coordinated by a series of transcriptional factors, including members of the nuclear receptors family, such as liver X receptor (LXR) and farnesoid X receptor (FXR) as well as the sterol regulatory element binding proteins (SREBPs) [14].

## 2. The Nuclear Receptors

Nuclear receptors (NRs) are a major component of signal transduction in animals. They are metabolite- and hormone-sensing transcription factors that translate dietary or endocrine signals into changes in gene expression. They have been described as modulators of not only many hormone activities, but also important nutrients and metabolites involved in the homeostasis and physiology of cells and tissues [15].

The NR superfamily contains transcriptional regulators that are conserved throughout metazoans, including nematodes, insects, and vertebrates [16]. For example, there are 48 and 49 NR members encoded in the human and mouse genome, respectively. NRs can bind their DNA target sites as a monomer (e.g., steroidogenic factor (SF-1)), homodimer (e.g., estrogen receptor (ESR)), or heterodimer (e.g., FXR and LXR form heterodimers with the retinoid X receptor RXR). NRs can be ligand-dependent or ligand-independent transcription factors that activate or repress gene expression [17]. They play important roles in diverse functions such as homeostasis, reproduction, development, inflammation, toxicology, and metabolism [18]. NRs are thus key players in the regulation of complex gene networks.

The known endogenous ligands for NRs consist of a wide range of chemical structures, such as bile acids, phospholipids, steroid hormones, thyroid hormone, retinoids, and vitamin D [19]. It is interesting to note that many of these ligands are derived from cholesterol, suggesting that NRs have an important role in cholesterol-related metabolism and pathology. Additionally, it has been suggested that one and the same NR may have distinct endogenous ligands in different tissues or cell types [20]. This could be particularly relevant to design therapeutic interventions selectively targeting the availability of one ligand without interfering with the desired effects of another.

**Figure 1:**
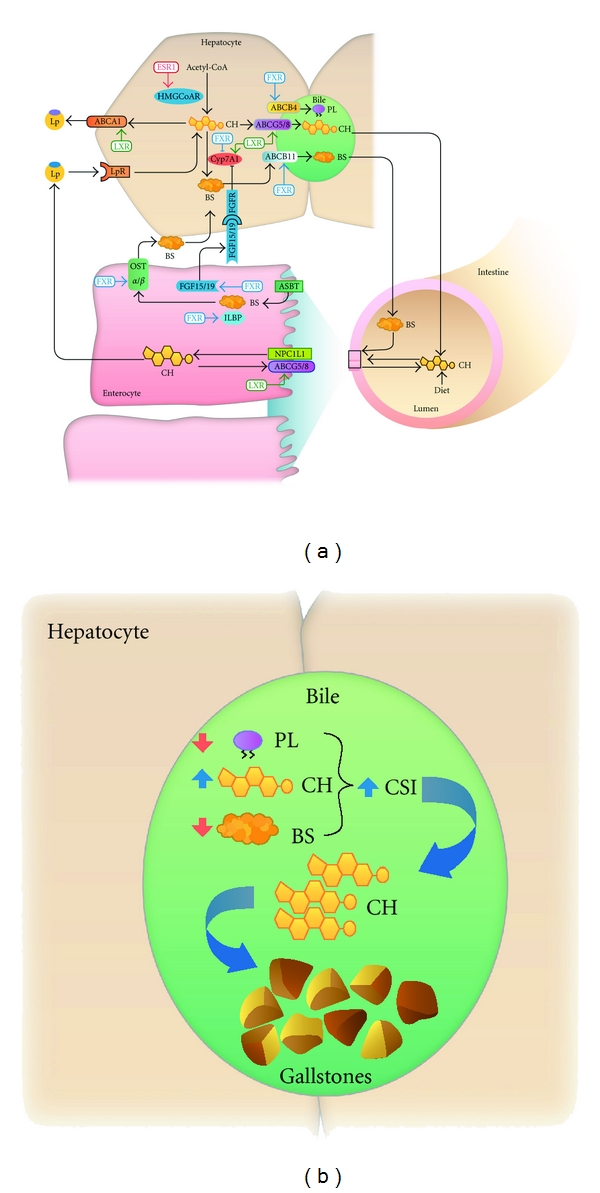
(a) Possible molecular mechanisms of action of nuclear receptors at the liver and the small intestine. Cholesterol derived from the diet as well as from the bile enters the intestine and is absorbed by the enterocytes through NPC1L1 and can be secreted back to the intestinal lumen by ABCG5/G8. After absorption, cholesterol is incorporated into lipoproteins (Lps), secreted into lymph and blood, and transported to the liver after triglyceride uptake in peripheral tissues. Bile salts (BSs) are absorbed in the intestine by the ASBT transporter and exit into the basolateral surface through OST*α*/*β* transporters, among others, reaching the liver via the systemic blood circulation. The hepatic pool of cholesterol originates from *de novo* synthesis from acetyl-CoA as well as receptor-mediated endocytosis and/or selective lipid uptake from Lp. Cholesterol can be secreted into plasma HDL through ABCA1 transporter or by formation and secretion of VLDL (not shown) or into the bile through the heterodimeric ABCG5/8 transporter. Bile is constituted by cholesterol (CH), phospholipids (PLs), and BSs. PL enters the biliary canaliculi through the ABCB4 transporter. BSs, obtained by neosynthesis from cholesterol or by uptake from plasma, are secreted into the bile by the ABCB11 transporter. The NRs control metabolism and secretion of lipids at different levels: LXR promotes cholesterol efflux from the intestine and from the liver by activation of ABG5/8 and ABCA1 transporters. Also, LXR activates Cyp7A1 leading to an increase in BS synthesis in the liver. The FXR receptor regulates BS concentration at two different levels: promoting the expression of FGF15/19, ILBP, and OST*α*/*β* transporters in the intestine as well as increasing the expression of ABCB4 and ABC11 transporters and repressing Cyp7A1 expression in the liver. ESRs increase *de novo* cholesterol synthesis by regulation of HMGCoAR.ASBT: apical sodium bile acid transporter. OST*α*/*β*: organic solute transporter alpha/beta (b) Cholesterol gallstone formation. An increase in cholesterol and/or a decrease in BS or PL contents in the bile lead to an increase in the biliary cholesterol saturation index (CSI) triggering cholesterol precipitation into crystals and ultimately the formation of cholesterol stones within the gallbladder.

This paper summarizes some recent progress in understanding the role of some NRs, including heterodimeric LXR and FXR and homodimeric ESR, on biliary lipid secretion and their potential clinical implications for CGD. The principal features of mechanisms underlying the effect of NRs on liver and intestine lipid metabolism and transport and CGD are depicted in Figures 1(a) and 1(b).

## 3. The Liver X Receptor 

The liver X receptors (LXRs), LXR*α* and LXR*β*, are oxysterol intracellular sensors that regulate key genes related to sterol, bile acid, and lipid homeostasis [21, 22]. In rodents, but not in humans, LXR promotes bile acid synthesis by activating the expression of *Cyp7A1, *the limiting enzyme of the neutral bile acid synthesis pathway [23–25]. LXRs are also known to induce the hepatic expression of cholesterol and phospholipid efflux transporters, including canalicular ABCG5/ABCG8 [26] as well as ABCA1, a basolateral ABC transporter of cholesterol and phospholipids [27]. 

Uppal et al. evaluated the effect of hepatic LXR activation on lithogenic-diet-fed transgenic mice with constitutively active expression of LXR [28]. They found an increased susceptibility of these mice to gallstone disease that correlated with increased biliary concentrations of cholesterol and phospholipids and decreased biliary bile salt concentrations, leading to a high cholesterol saturation index in bile. As expected, hepatic expression of the canalicular transporters *Abcg5/Abcg8 *was induced, as well as *Abca1* and *Cyp7A1*, by administration of LXR agonists in lithogenic-diet-fed LXR transgenic mice. Moreover, the prolithogenic effect of LXR activation was abolished in low-density-receptor-deficient mice. On the other hand, ezetimibe, a cholesterol-lowering agent that blocks intestinal cholesterol absorption, had the same effect. These results confirm that hepatic LDL cholesterol uptake and intestinal cholesterol absorption are relevant for gallstone disease in this specific diet-induced gallstone disease mouse model.

In humans, increased expression of LXR, ABCG5, and ABCG8 was found in livers of nonobese Chinese gallstone patients. Moreover, increases in mRNA levels of these genes significantly correlated with biliary cholesterol levels and saturation [29], suggesting a potential pathogenic role of LXR activation in human gallstone disease. 

Genomewide analysis of gallstone traits in inbred mouse strains has yielded a susceptibility map of lithogenic (Lith) loci [30–33]. Interestingly, the Lith1 locus harbors LXR*α* as a candidate gene in addition to ABCA11 [33]. However, no evidence of association between single nucleotide polymorphisms (SNPs) for the LXR gene and gallstone susceptibility was detected in a German population sample [34]. Clearly, further studies are required to elucidate the relevance of this hepatic nuclear receptor in the pathogenesis of this disease in humans.

Although studies evaluating the relevance of intestinal LXR in gallstone disease are lacking, intestine-specific LXR activation decreased cholesterol absorption in transgenic mice with intestinal expression of constitutively active LXR [35]. This phenotype correlated with upregulation of the *Abcg5/Abcg8* transporters, which are localized in the apical membrane in the intestine and mediate cholesterol efflux [26]. Indeed, these transgenic mice fed with a high-cholesterol diet were protected against hepatic cholesterol accumulation. Thus, in contrast to hepatic LXR activation, it could be speculated that intestinal LXR activation would protect from CGD. This opens a window for future therapeutic interventions, directed to selective LXR activation in the intestine, avoiding the side effects of hepatic LXR stimulation, such as increased liver and plasma triglyceride levels.

## 4. The Farnesoid X Receptor 

The farnesoid X receptor (FXR) acts as an intracellular bile salt sensor [36, 37], induces the expression of ABCB11 and ABCB4, and represses bile salt synthesis by small-heterodimer-partner-(SHP-) mediated* Cyp7A1* inhibition [36–38]. FXR was also identified as an attractive candidate gene for gallstone disease in mice by genomewide investigation studies [32]. Moreover, lower expression of *Fxr* was observed in a mouse strain susceptible for gallstone formation in comparison with a resistant strain [32]. In addition, mice with isolated hepatic insulin resistance and increased gallstone susceptibility exhibited increased bile salt hydrophobicity in bile and partial resistance to FXR activation by GW4064, a synthetic FXR agonist [39]. More striking, Moschetta et al. [40] found that FXR deficiency in mice conferred a higher susceptibility to CGD when fed a lithogenic diet. This increased susceptibility correlated with a higher bile salt hydrophobicity index and gallbladder mucosal inflammation. Also, they found a decreased expression of the ABCB4 and ABCB11 transporters involved in biliary phosphatidylcholine and bile salt secretion. In addition, treatment of lithogenic-diet-fed gallstone-susceptible mice with FXR agonist GW4064 prevented cholesterol gallstone formation and increased the expression of ABCB11 and ABCB4 transporters, resulting in substantially higher bile salt and phospholipid bile concentrations in gallbladder bile. These results suggest that modulation of FXR and their downstream targets may be a good strategy for drug therapy in human CGD; as well as the modulation of other nuclear receptors has been used in several other human pathologies [41]. Pharmacological activation of FXR can selectively increase the secretion of bile salts and phospholipids, by increasing expression of the ABCB11 and ABCB4 transporters, allowing the solubilization of cholesterol in bile.

Some studies in humans have also supported a role of FXR in gallstone disease. Kovacs et al. showed an association of a sequence variant in the FXR gene with gallstone prevalence in a Mexican cohort [42]. However, no relationship of this SNP with gallstones was detected in a German cohort, whereas a trend toward a protective effect of the same SNP was found in a Chilean population. Interestingly, FXR variants have been found in Caucasian patients with intrahepatic cholestasis of pregnancy, a condition known to be associated with gallstones [43]. In addition, a small study described the association between reduced hepatic expression of the PPAR-*γ* coactivator-1 (PGC-I) and decreased FXR levels in gallstone patients [44]. Based on this finding as well as the role of PGC-1 as a positive activator of FXR expression [45], the authors speculated that PGC-1 may function as a protective gene for gallstone disease by increasing FXR activity. In summary, current data strongly suggest a relevance of FXR in human gallstone disease point, but more studies are still required to fully validate this hypothesis. 

Besides its role in hepatic lipid homeostasis, FXR activity should also be considered as a regulator of lipid genes expressed in the intestine. In this regard, decreased intestinal expression of FXR and its target genes, ileal lipid-binding protein (ILBP) and OST*α*–OST*β* (all involved in bile acid transport), has been described in a subgroup of nonobese gallstone female patients [46, 47]. These findings suggest a FXR-dependent defect in the intestine leading to decreased bile acid absorption and subsequently diminished bile acid pool. Accordingly, increased bile acid and cholesterol synthesis have been reported in a subgroup of Chilean patients [48], suggesting that increased intestinal loss of bile acids may precede gallstone formation.

Another interesting FXR gene target is the fibroblast growth factor (FGF) 15/19 (mouse and human ortholog, resp.). FXR induced the expression of FGF15, which activated a negative feedback on hepatic bile acid neosynthesis after binding to FGF receptor 4 and impaired gallbladder emptying after binding to FGF receptor 3 [49]. Interestingly, ileal FGF19 mRNA levels were diminished in nonobese gallstone females compared with controls [47]. Further studies are required to elucidate if FGF19 has a direct role in the pathogenesis of CGD.

## 5. Estrogen Receptors

As it is well documented by epidemiological and clinical studies, CGD prevalence is higher in women than in men [50–52]. Physiological increase of estrogen levels, in conditions such as human pregnancy, correlates with increased hepatic secretion of biliary cholesterol and the formation of a cholesterol-supersaturated bile [53]. Furthermore, oral contraceptive steroids and conjugated estrogens increase the risk for CGD [54–56]. Interestingly, estrogens exert their biological functions through the modulation of two closely related classical homodimeric nuclear receptors, ESR1 and ESR2, which are widely expressed in tissues, including the liver [57–59]. Together, these data have lead to the hypothesis that estrogens may enhance the risk for CGD by increasing the functions of the hepatic ESRs [2].

Using gonadectomized gallstone-resistant male or female AKR mice fed with a lithogenic diet in the presence of ESR-selective synthetic estrogens has shown a correlation between gallstone formation and hepatic ESR1 upregulation. Furthermore, the prolithogenic action of estrogens was blocked by ESR1-selective antagonists, suggesting that ESR1 is the specific estrogen receptor pathogenically linked to gallstone formation. Increased gallstone formation mediated by estrogen administration in this animal model correlated with higher biliary cholesterol secretion and the presence of cholesterol supersaturated bile [59].

High plasma levels of estrogens have been correlated with augmented activity of the cholesterol biosynthesis rate-limiting enzyme HMG-CoA reductase in humans and animals [60, 61], even under high-cholesterol diets. Wang et al. studied the relevance of hepatic cholesterol neosynthesis for estrogen-induced gallstone formation in AKR ovariectomyzed mice treated with estrogens and fed with chow or high-cholesterol diets [61]. They found that estrogens induced an increase in cholesterol biosynthesis, even in the presence of a high cholesterol diet. These changes correlated with increased expression of SREBP2, the key transcription factor regulator of the HMG-CoA reductase gene, and also its target genes [61]. There was also an augmented biliary cholesterol secretion, with an important increase in the contribution of newly synthesized cholesterol to biliary cholesterol output. Consistent with accelerated gallstone formation, a higher lithogenicity of the bile was found. Moreover, estrogens could also act at the canalicular membrane by increasing ABCG5/ABCG8 activity [2]. These results have led to a model in which estrogen induces cholesterol gallstone formation by promoting cholesterol biosynthesis through SREBP2 and hepatic biliary cholesterol secretion. 

On the other hand, estrogens can also regulate lipid and bile salt metabolism through GPR30 receptor activation. This novel estrogen receptor, a member of the rhodopsin-like family of G-protein-coupled receptors, is a multipass membrane protein that has been found in the endoplasmic reticulum and the cell surface. In normal physiological conditions, GPR30 is widely expressed, with particularly high expression reported in heart, lung, liver, intestine, ovary, and brain [62]. This pattern of expression leads us to propose a possible metabolic role of GPR30 activation not only in the liver, but also in the small intestine as documented for LXR and FXR receptors. In this regard, estrogen activation of GPR30 may influence CGD through nongenomic activation of rapid kinase signalling pathways. 

## 6. Concluding Remarks

In the past few years, significant advances have been made in understanding the possible molecular mechanisms that link some nuclear receptors such as LXR, FXR, and ESRs with CGD. In the liver as well as in the small intestine, these receptors regulate the expression of key genes involved in synthesis and transport of cholesterol, bile salts, and phospholipids. In such a way, nuclear receptors may modulate bile lipid composition and thus the susceptibility to cholesterol gallstone formation. Even though new insights have been obtained using animal models, more studies are needed to establish more definitively their relevance in human CGD.

The knowledge of nuclear-receptor-dependent mechanisms involved in CGD opens a new opportunity for drug therapy of this disease condition based on modulation of hepatic and/or intestinal cholesterol and bile acid metabolism. Modulation of intestinal lipid metabolism by nuclear receptors as well as the role of estrogen receptors must be explored more deeply to offer new targets for drug development on CGD. In this regard, therapeutic approaches to CGD would not be limited to the classically liver-related receptors LXR and FXR.
